# Altered hormonal milieu and dysregulated protein expression can cause spermatogenic arrest in ectopic xenografted immature rat testis

**DOI:** 10.1038/s41598-019-40662-y

**Published:** 2019-03-11

**Authors:** Sandeep Goel, Naojiro Minami

**Affiliations:** 1 Laboratory of Reproductive Biology, Department of Applied Biosciences, Graduate School of Agriculture, Kyoto University, Kyoto, 606-8502 Japan; 2 Laboratory for the Conservation of Endangered Species, Centre for Cellular and Molecular Biology, Council for Scientific and Industrial Research, Uppal Road, Hyderabad, 500 007 India

## Abstract

Testis tissue xenografting complemented with cryopreservation is a feasible technique for fertility preservation in children with malignancy receiving gonadotoxic therapy and for endangered species with high neonatal mortality rate. However, xenografted testis of human and most endangered species are known to undergo spermatogenic arrest. In this study, we xenografted immature rat testis onto immunodeficient male mice to investigate the plausible underlying causes of spermatogenic arrest. Histological analysis of xenografted testes collected 8-wk post-grafting showed incomplete spermatogenesis with pachytene-stage spermatocytes as the most advanced germ cells. Although the levels of serum luteinizing hormone and testosterone were normal in recipient mice, those of follicle stimulating hormone (FSH) were significantly high, and specific receptors of FSH were absent in the xenografts. The xenografts demonstrated dysregulated expression of Sertoli cell-transcriptional regulators (WT1 and SOX9) and secretory proteins (SCF and GDNF). In conclusion, results from our study suggested that an altered hormonal milieu in recipients and dysregulated protein expression in xenografts could be a potential cause of spermatogenic arrest in xenografted immature rat testis. Further stereological analysis of xenografts can demonstrate precise cellular composition of xenografts to decipher interactions between germ and somatic cells to better understand spermatogenic arrest in xenografted testis.

## Introduction

Ectopic testis tissue xenografting offers a practical method for understanding the mechanism of spermatogenesis and testicular maturation. This technique has been used for the production of mature gametes by grafting small pieces of testis tissue under the dorsal skin of immunodeficient mice recipients^1^. Xenografting complemented with cryopreservation of testis tissue can find application in fertility preservation in children with malignancy receiving gonadotoxic treatment and in the conservation of endangered animals with high neonatal mortality rate. Production of live offspring from cryopreserved-xenografted testis of rabbit^2^, and more recently from pig^3^, showed the applicability of these techniques. Of the 23 species of mammals used as donors for testicular tissue xenografting to date, 15 showed complete spermatogenesis^1^. In the remaining 7 species, including endangered ungulates (Banteng^4^, Mohor gazelle, Cuvier’s gazelle^5^), Iberian lynx^5^ (an endangered feline), common marmoset^6^, laboratory rat^7–9^ and humans^7,10–13^, spermatogenic arrest occurred at the spermatogonia, spermatocyte, or round spermatid stages. We recently showed that spermatogenesis was incomplete and arrested at the spermatocyte stage in xenografted testis from an endangered ungulate (Indian spotted mouse deer)^14^. It is known that genetics, hormonal, thermal, and toxic factors are implicated in spermatogenic arrest in humans^15^. Although exogenous gonadotropin treatment of the recipient mice was demonstrated to aid completion of spermatogenesis in xenografted testis of certain species, these results were inconsistent^6,16,17^. Therefore, the key factors that lead to spermatogenic arrest in xenografted testis still remain unclear, and the underlying mechanism needs to be investigated to increase the efficiency of xenografting. Further insights into spermatogenic arrest in xenografts would help in developing methods to overcome it.

Normal spermatogenesis and fertility are dependent upon paracrine interactions between the somatic cells and the germ cells, and endocrine support from the pituitary gland^18^. Development and differentiation of germ cells require the interaction of germ cells with the supporting Sertoli cells in the epithelium of the seminiferous tubules. Failure of germ cell–somatic cell interactions could be one of the causes of spermatogenic arrest. Because rat testis xenografts show spermatogenic arrest^7–9^, they can therefore serve as a suitable model for studying spermatogenic arrest. The purpose of the present study is to identify the factors that play a role in spermatogenic arrest using the rat-to-mouse testis xenograft model by evaluating endocrine changes in the recipients, and changes in protein expression in xenografts.

## Results

### Xenograft and seminal vesicle weights, and hormonal assay

**Figure 1:**
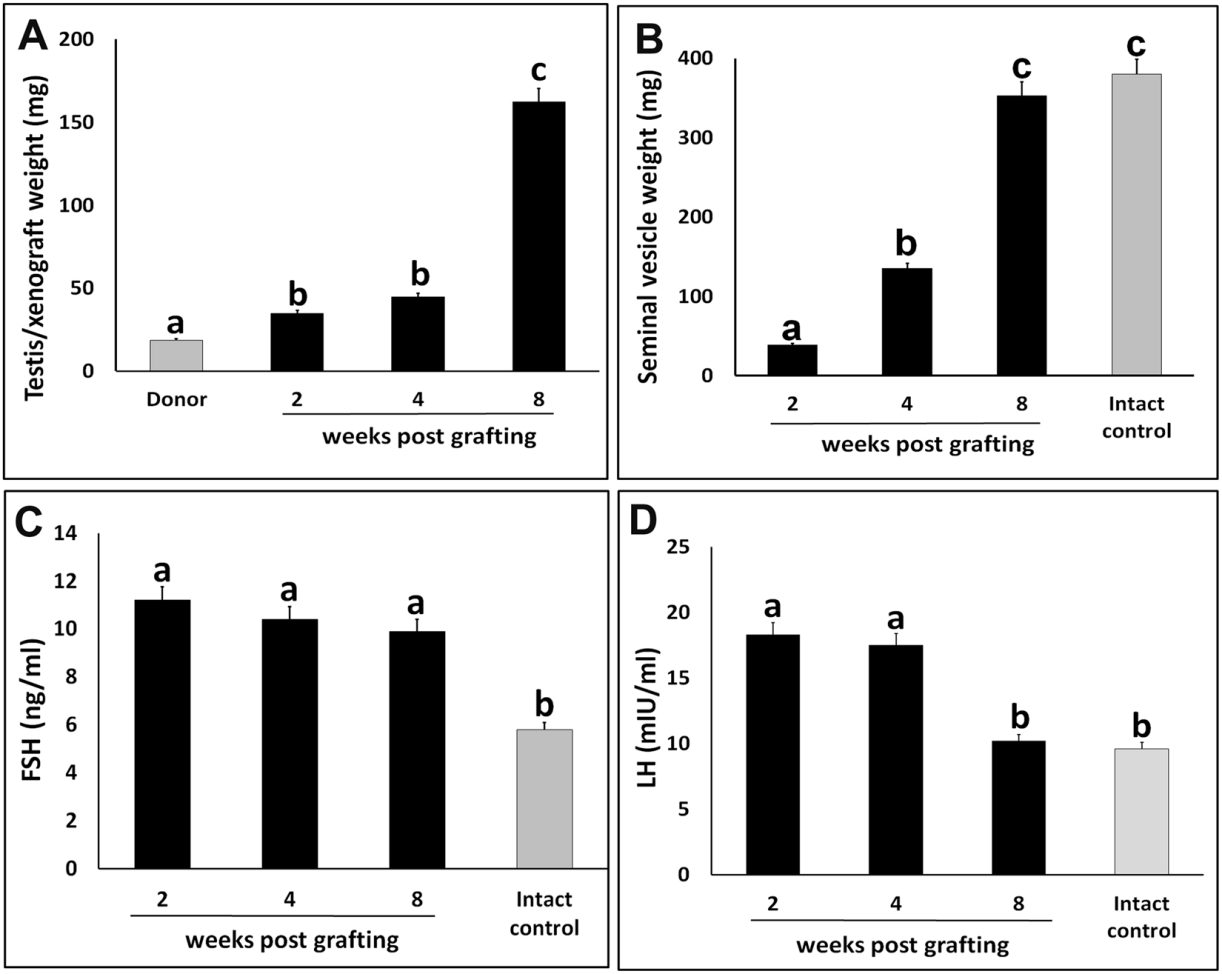
Assessment of xenograft growth and hormonal status of recipient mice. (**A**) Donor testis and average xenograft weight at 2-, 4-, and 8-wk post-grafting. (**B**) Average castrated seminal vesicle weight of recipient mice at 2-, 4-, and 8-wk post-grafting. (**C**) Average serum FSH and (**D**) LH levels of recipient mice at 2-, 4-, and 8-wk post-grafting. Seminal vesicle weight, and serum FSH and LH levels of intact male mice were used for comparison. Data are presented as mean ± SEM. Bars with different letters are significantly different at P < 0.05.

At 2-, 4-, and 8-wk post-grafting, xenografts and seminal vesicles were recovered from recipients and weighed (Table 1, Fig. 1A,B). Follicle stimulating hormone (FSH) and luteinizing hormone (LH) levels were estimated in the recipient’s blood (Fig. 1C,D). The xenograft recovery did not differ significantly at 2-, 4-, and 8-wk post-grafting (Table 1; P < 0.05). The weight of the xenografts increased around 3-fold at 2-wk post-grafting (Fig. 1A; P < 0.05). The increase in the weight of the xenografts collected at 4 wk was not significant compared with that of those collected at 2 wk (P > 0.05). However, the weight of the xenografts significantly increased 8-fold at 8-wk post-grafting (P < 0.05). Similarly, a significant increase in the weight of the seminal vesicles in the recipient was noted over 8 wk (Fig. 1B; P < 0.05). Seminal vesicle weight increased 3-fold at 4 wk and 9-fold at 8 wk when compared with that at 2 wk. At 8-wk post-grafting, the seminal vesicle weight of the recipient was comparable to that of the intact control mice (P > 0.05).

**Table 1:** Experimental graft data for testis xenografted and collected 2-, 4-, and 8 wk post-grafting.

Collection time post-grafting	Number of mice grafted and analyzed	Number of grafts recovered^a^ (%)	Number of grafts with meiotic germ cells^b^ (%)
2 wk	5	100 (20/20)	ND
4 wk	5	100 (20/20)	ND
8 wk	5	95.0 (19/20)	89.4 (17/19)

^a^Total xenografts removed divided by the total number of xenografts grafted.

^b^Percentage of recovered xenografts with pachytene-stage spermatocytes.

*Significantly different within the column (P < 0.05).

ND- Not determined.

Serum gonadotropins FSH and LH levels were raised in the recipients at 2- and 4-wk post-grafting (Fig. 1C,D; P < 0.05). Although LH level in the recipients was comparable with that of the intact control mice (P > 0.05) at 8-wk post-grafting, FSH level remained significantly high (P < 0.05).

### Histological evaluation

**Figure 2:**
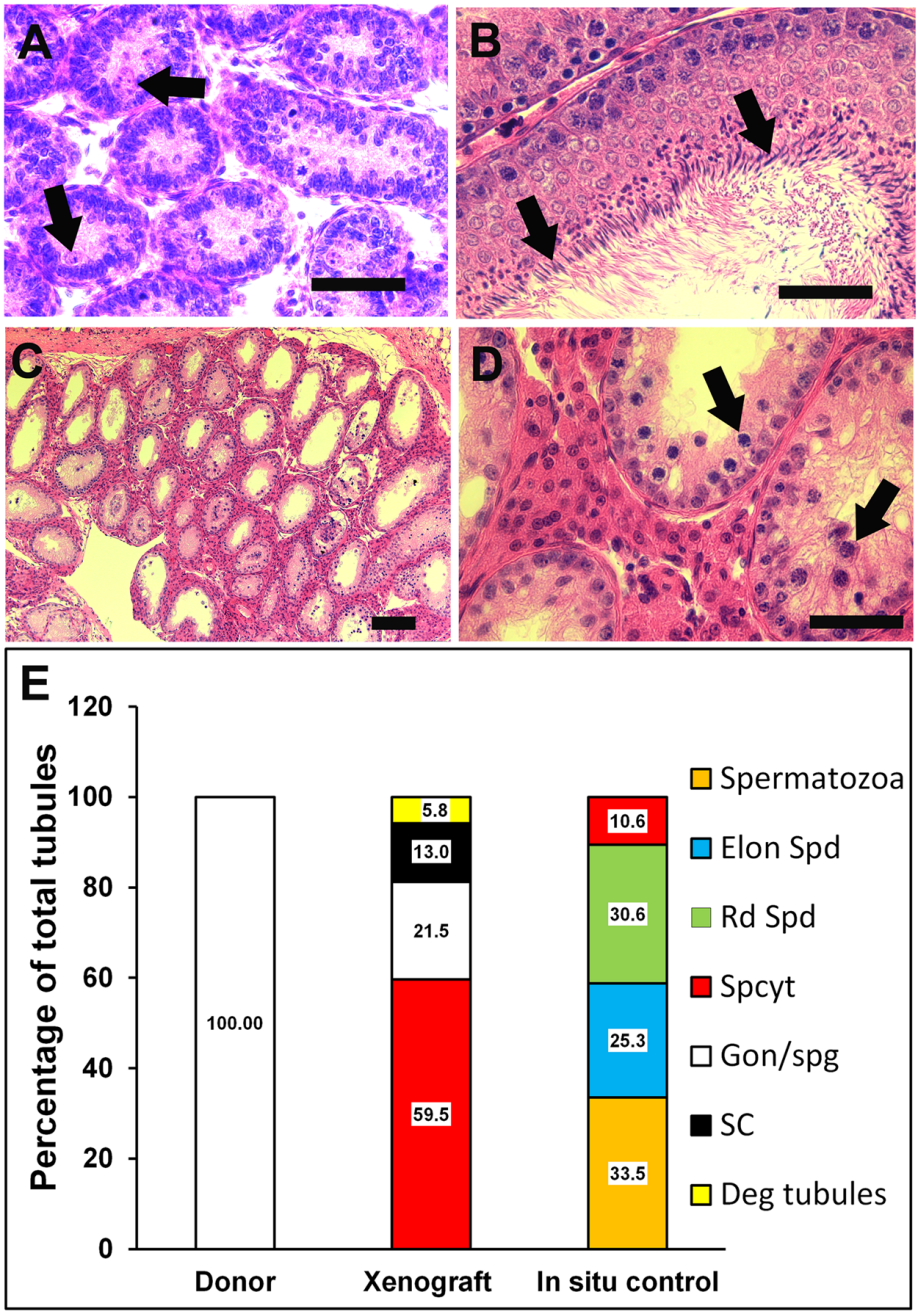
Histological examination and quantitative assessment of seminiferous tubules for the most advanced germ cell type. (**A**) An immature donor tissue from a 7-day-old rat. Note that the most advanced germ cells at this age were gonocytes/spermatogonia (arrows). (**B**) A 9-wk-old *in situ* control testis. Note that the most advanced germ cells identified were spermatozoa (arrows). (**C**) Low and (**D**) high magnification image of a xenograft collected at 8-wk post-grafting. Note that the most advanced germ cells identified in the xenografts were pachytene-stage spermatocytes (**D**, arrows). (**E**) Percentage of seminiferous tubules with the most advanced germ cell type. Deg tubules, degenerated tubules; SC, Sertoli cell only; Gon/spg, gonocytes or spermatogonia; Spcyt, pachytene spermatocytes; Rd Spd, round spermatid; Elon Spd, elongated spermatid; Spermatozoa, spermatozoa. Scale bar = 50 µm.

Histological evaluation of 7-day-old rat donor testis showed seminiferous tubules with spermatogonia as the most advanced germ cell in all the tubules (Fig. 2A,E). Testis from adult male rats (9 -wk-old) used as *in situ* control showed complete spermatogenesis, with spermatozoa as the most advanced germ cells (Fig. 2B,E). Xenografted testis from donors could establish spermatogenesis following ectopic xenografting, and meiosis was induced, as indicated by the presence of pachytene-stage spermatocytes as the most advanced germ cells (Fig. 2C–E).

### Western blot analysis

**Figure 3:**
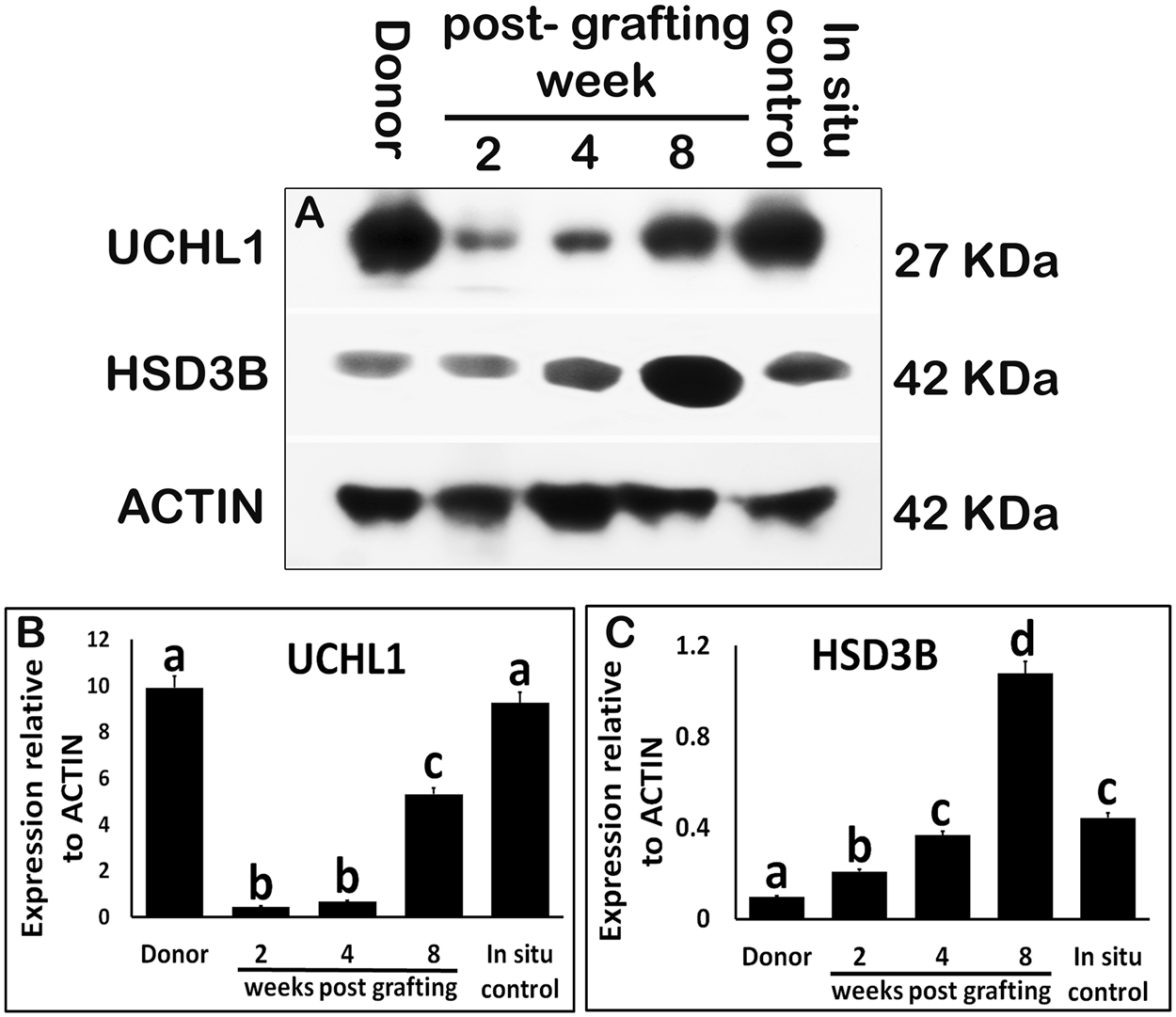
Western blot analysis of xenografted testes at 2-, 4-, and 8-wk post-grafting for expression of spermatogonia and Leydig cell-specific proteins. Protein expression in 7-day-old donor testes (donor) before grafting is presented as starting material and 9-week-old rat testis as *in situ* control. (**A**) Representative blot and densitometry analysis of (**B**) UCHL1 and (**C**) HSD3B protein. Y-axis represents the intensity of bands relative to ACTB. Data are presented as mean ± SEM. Bars with different letters are significantly different at P < 0.05.

Xenografts collected at 2-, 4-, and 8-wk post-grafting, donor, and *in situ* control testes were analyzed for the expression of germ and somatic cell-specific proteins. The expression of ubiquitin carboxy-terminal hydrolase L1 (UCHL1), a spermatogonia-specific protein, was low in xenografts at 2- and 4-wk post-grafting when compared with that in donor and *in situ* control testis (Fig. 3A,B; P < 0.05). However, at 8-wk post-grafting, UCHL1 expression increased significantly when compared with that at 2- and 4-wk post-grafting (P < 0.05). In spite of the upsurge, the expression level was still lower than that found in the donor and *in situ* testis (P < 0.05).

Expression of 3 beta-hydroxysteroid dehydrogenase (HSD3B) protein progressively increased in xenografts, by 2 folds at 2 wk and 4 folds at 4 wk, compared with that of the donor testis (Fig. 3A,C, P < 0.05). However, HSD3B expression peaked at 8 wk and was almost 2.5-fold higher than that *in situ* control testis (P < 0.05).

**Figure 4:**
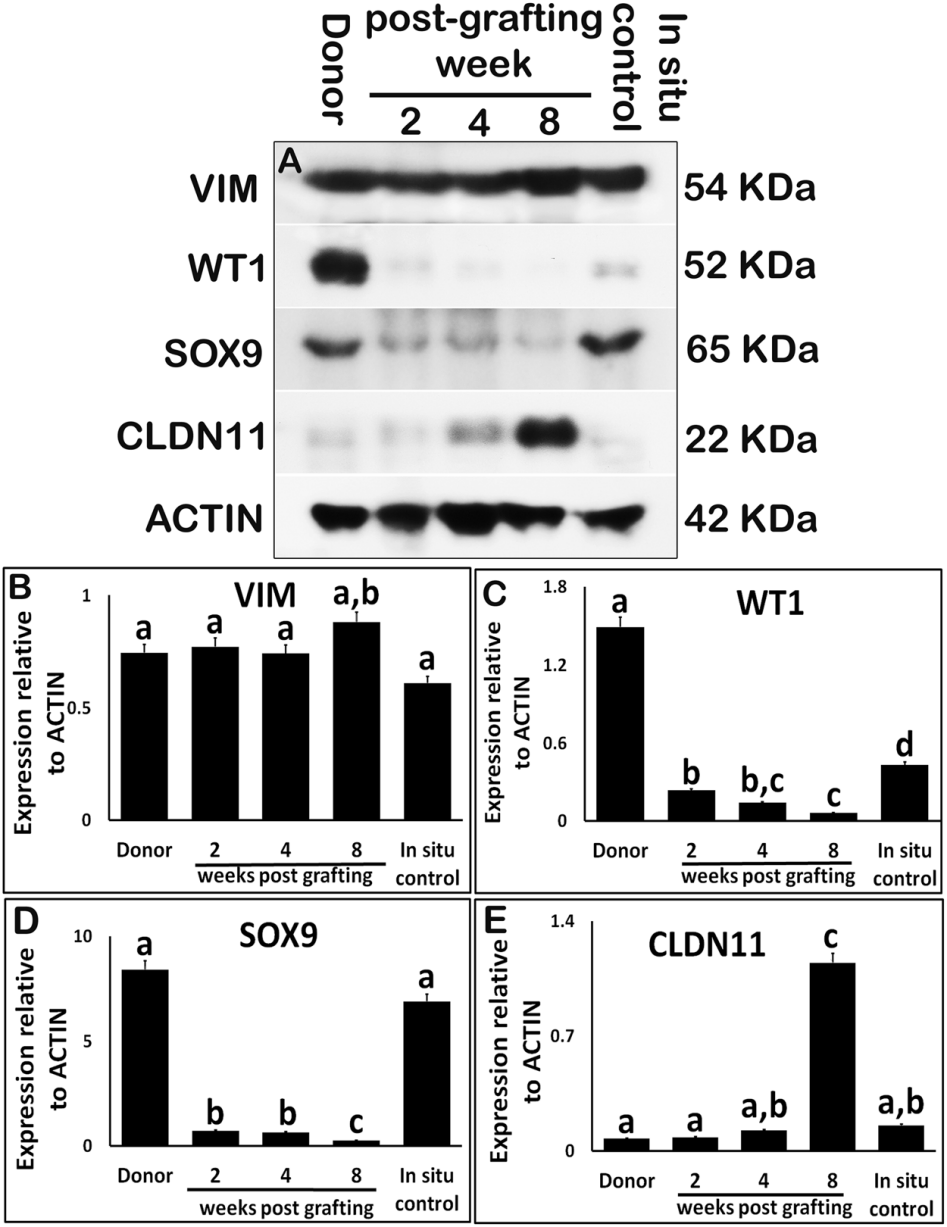
Western blot analysis of xenografted testis at 2-, 4-, and 8-wk post-grafting for expression of Sertoli cell structural, transcription regulators, and tight junction transmembrane proteins. Protein expression in 7-day-old donor testes (donor) before grafting is presented as starting material and 9-week-old rat testis as *in situ* control. (**A**) Representative blot and densitometry analysis of (**B**) VIM, (**C**) WT1, (**D**) SOX9, and (**E**) CLDN11 protein. Y-axis represents the intensity of bands relative to ACTB. Data are presented as mean ± SEM. Bars with different letters are significantly different at P < 0.05.

Expression of the Sertoli cell-specific structural protein Vimentin (VIM) was comparable with that in the donor, *in situ* control testis, and xenografts at all collection time points (Fig. 4A,B; P > 0.05). Interestingly, the expression of VIM in xenografts at 8 wk was elevated but was not different from *in situ* control testis (Fig. 4B; P > 0.05). However, expression of Sertoli cell-specific transcription regulators such as Wilms tumour 1 (WT1) and SRY-box 9 (SOX9) was significantly downregulated in the xenografts (Fig. 4A,C,D; P < 0.05) and was the lowest at 8 wk.

Claudine 11 (CLDN11) expression in xenografts at 2- and 4-wk post-grafting was not different from that in the donor or the *in situ* control testis (Fig. 4A,E; P > 0.05). However, CLDN11 expression was significantly elevated in xenografts at 8-wk post-grafting (P < 0.05).

**Figure 5:**
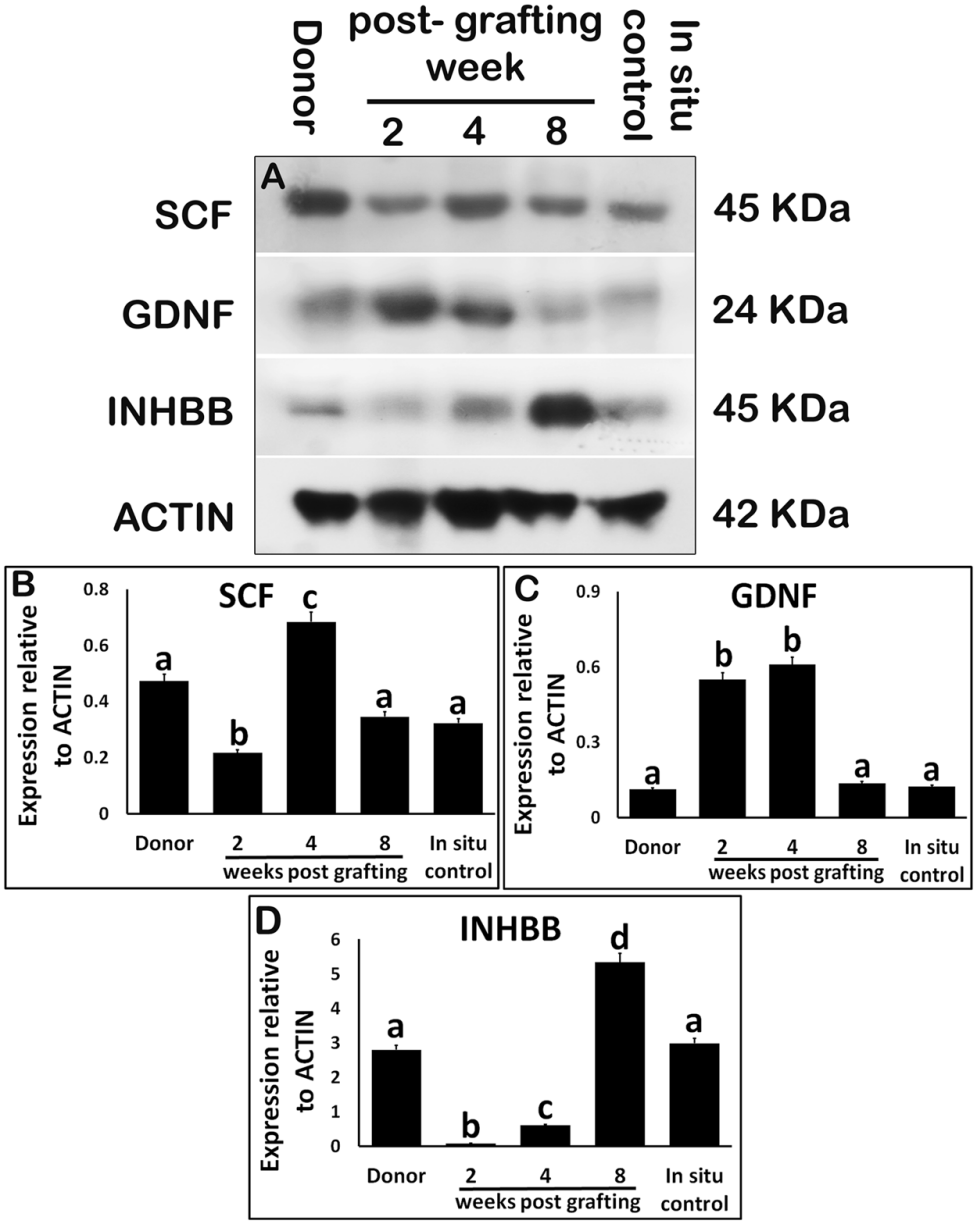
Western blot analysis of xenografted testis at 2-, 4-, and 8-wk post-grafting for expression of Sertoli cell secretory proteins. Protein expression in 7-day-old donor testes (donor) before grafting is presented as starting material and 9-week-old rat testis as *in situ* control. (**A**) Representative blot and densitometry analysis of (**B**) SCF, (**C**) GDNF, and (**D**) INHBB protein. Y-axis represents the intensity of bands relative to ACTB. Data are presented as mean ± SEM. Bars with different letters are significantly different at P < 0.05.

**Figure 6:**
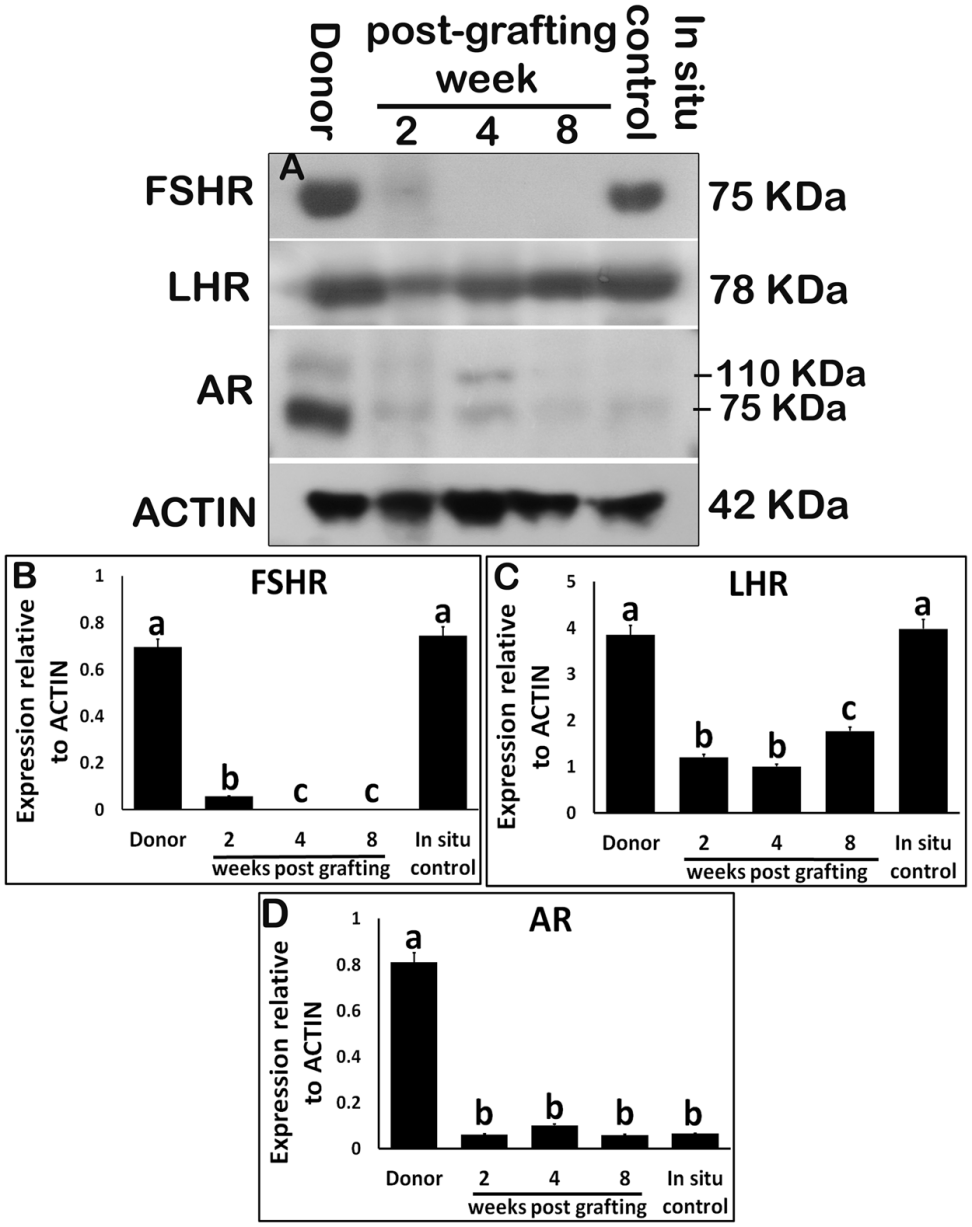
Western blot analysis of xenografted testis at 2-, 4-, and 8-wk post-grafting for expression of hormone receptors. Protein expression in 7-day-old donor testes (donor) before grafting is presented as starting material and 9-week-old rat testis as *in situ* control. (**A**) Representative blot and densitometry analysis of (**B**) FSHR, (**C**) LHR, and (**D**) AR protein. Y-axis represents the intensity of bands relative to ACTB. Data are presented as mean ± SEM. Bars with different letters are significantly different at P < 0.05.

Expression of stem cell factor (SCF) was low in xenografts at 2-wk post-grafting (Fig. 5A,B; P < 0.05). However, elevated expression was evident at 4 wk (P < 0.05). Expression of glial cell-derived neurotrophic factor (GDNF) protein was elevated in xenografts at 2- and 4-wk post-grafting (Fig. 5A,C; P < 0.05). Interestingly, the expression of SCF and GDNF in xenografts at 8-wk post-grafting was comparable with that in donor and *in situ* control testis (P > 0.05). Inhibin beta b subunit (INHBB) level was lower in xenografts collected at 2- and 4-wk post-grafting when compared with that in donor and *in situ* control testis (Fig. 5A,D; P < 0.05). However, INHBB level was significantly elevated (P < 0.05) in xenografts at 8-wk post-grafting,

The expression of FSH receptor (FSHR) decreased significantly in xenografts at 2-wk (Fig. 6A,B; P < 0.05) and was absent at 4- and 8-wk post-grafting. Contrary to FSHR, LH receptor (LHR) was expressed in xenografts at all collection time points. Xenografts collected at 2-wk post-grafting had significantly lower LHR expression compared with that in donor and *in situ* control testis, and it remained low until 4 wk (Fig. 6A,C; P < 0.05). However, LHR expression rose significantly at 8 wk (P < 0.05) but remained lower than that in donor and AMC testis (P < 0.05). Although elevated expression of androgen receptor (AR) was evident in donor testis, expression of AR in xenografts was not different from that in the *in situ* control testis post-grafting (Fig. 6A,D; P > 0.05).

## Discussion

**Figure 7:**
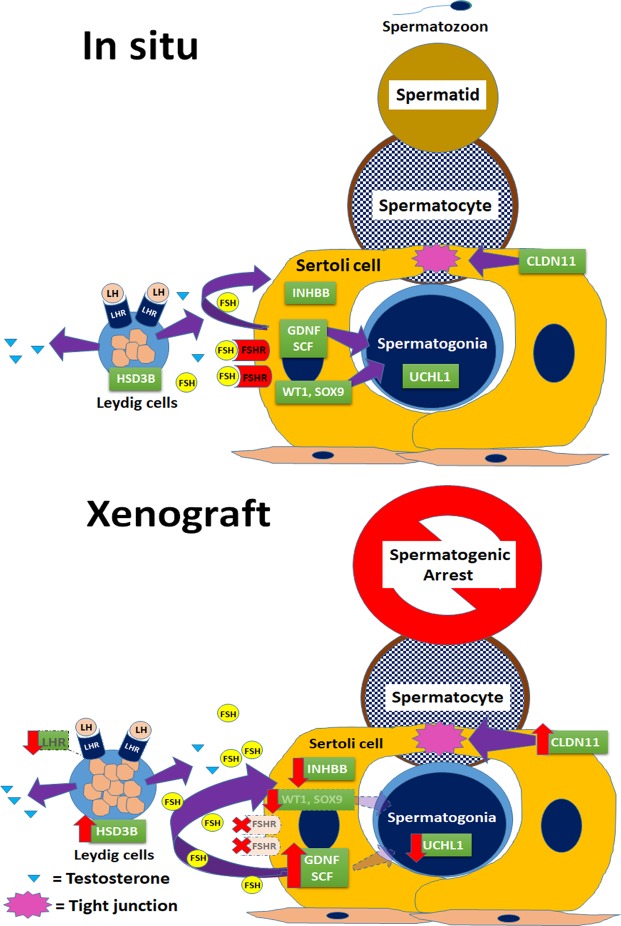
A model for spermatogenic arrest in xenografted testis. Sertoli cell secretory factors (GDNF, SCF, and INHBB), tight-junction transmembrane protein (CLDN11), and transcription regulators (WT1 and SOX9) expression create an intricate balance, which leads to proliferation and differentiation of spermatogonia (marked by UCHL1 expression). The gonadotropins FSH and LH play a critical role in the progression of spermatogenesis via their respective receptors (FSHR and LHR). Testosterone production in Leydig cells is regulated by LH, which controls steroidogenesis. In xenografts, elevation of SCF and GDNF expression leads to survival and proliferation of Sertoli cells but not spermatogonia, leading to loss of spermatogonia, as indicated by downregulation of UCHL1 expression. Downregulation of WT1 and SOX9 and elevation of CLDN11 indicate dysregulation of Sertoli cell-specific protein expression. An elevated serum FSH level of recipients and loss of FSHR from xenografts specifies disruption in the endocrine milieu. Dysregulation of INHBB expression may have caused an elevated FSH level. Although downregulation of LHR expression is apparent, LH and testosterone levels are restored in recipients. An elevated HSD3B level suggests hyperplasia of Leydig cell in xenografts responsible for the restoration of testosterone level in recipients.

*In situ*, germ and somatic cells communicate closely in the testis for the progression of spermatogenesis. An optimal hormonal milieu along with expression of cell-specific proteins, and transcription regulators create an intricate balance, which regulates testicular growth and maturation, and germ cell proliferation and differentiation to enable production of fertilization-competent gametes (Fig. 7). Following xenografting, immature rat testis showed a progressive increase in weight over 8 wk, suggestive of consistent growth of seminiferous tubules and supporting cellular structures^19^ and establishment of an effective vascular connection between the xenograft and the recipient, resulting in its survival^8^. Similarly, a progressive increase and subsequent restoration of the recipient’s seminal vesicle weight, an indicator of circulating bioactive testosterone^8,16,19–23^, suggests that not only the spermatogenic but also the steroidogenic function of the immature rat testis was recovered in the xenografts. This also indicates that the donor tissue responded to gonadotrophin stimulation of the recipient mice.

Histological evaluation of the xenografts collected at 8-wk post-grafting showed the presence of pachytene-stage spermatocytes as the most advanced germ cells, which suggests establishment of a synchronized hormonal interaction between the recipient’s pituitary and the xenografted immature rat testis that induced spermatogenesis. However, the absence of elongated spermatids in the xenografted testis indicated that the spermatogenesis remained incomplete. These results are consistent with previous reports in which incomplete spermatogenesis was reported in xenografted rat testis at 8-wk post-grafting with a spermatogenic arrest at pachytene-stage spermatocytes^8,9^. Spermatogenesis was observed to remain incomplete even after an extended grafting period (12 wk; data not shown). Since completion of spermatogenesis and appearance of mature sperm in rat testis *in situ* was reported 6-wk post-partum^24,25^, an inadequate grafting period could not be a likely cause of spermatogenic arrest in xenografts in our study.

In castrated recipients, gonadotropin levels were raised, which could be a result of a loss of inhibition by the testicular hormones, as observed in earlier studies using donor testis from neonatal mouse^21^ and pig^26^. The elevated gonadotropin levels likely stimulated Sertoli cell and Leydig cell proliferation, and maturation in xenografted testis. The restoration of serum LH levels to a level similar to that in the intact control mice suggests establishment of feedback loop between xenografts and recipient pituitary. However, failure to restore FSH level in recipients could be due to absence of a concomitant rise in inhibin level, because inhibin reportedly suppresses FSH secretion from the pituitary by a negative feedback loop^21,26^. Protein expression analysis of xenografts revealed that expression of INHBB level (a subunit of inhibin) was indeed low in xenografts collected at 2- and 4-wk post-grafting when compared with that in donor and *in situ* control testis. However, in xenografts collected at 8-wk post-grafting, INHBB level was significantly elevated. Although a constantly elevated FSH level may have caused an elevation in INHBB expression in xenografts at 8 wk, it was likely insufficient to lower the serum FSH level in the recipient. In earlier studies, an intermediate to elevated FSH level was reported in recipients of mice testis^21^; however, in recipients of pig testis, restoration of FSH level to a level similar to that in the intact control was reported to be due to elevated inhibin level^26^. However, spermatogenesis was completed in xenografts of mice and pig testis in both studies, unlike in the present study, where spermatogenesis remained incomplete in rat testis xenografts. This discrepancy could be because of the difference in the donor species.

Loss of FSHR from the xenografts could be attributed to sustained high serum FSH concentration in the recipients or to a higher sensitivity of rat Sertoli cells to elevated FSH level in castrated recipients. Interestingly, loss of FSHR from the xenografts did not seem to impair induction of spermatogenesis but likely prevented its completion. This finding is consistent with that in a previous study in which it was found that FSHR-deficient males were fertile but displayed small testes and partial spermatogenic failure^27^, indicating that spermatogenesis was initiated but not completed in the absence of FSHR. Because AR expression was unchanged in xenografts when compared to that in *in situ* control testis, it is likely that the AR-specific signaling may not have a significant role in spermatogenic arrest in the xenografts.

A low expression of spermatogonia-specific protein (UCHL1) in xenografts could be a result of loss of spermatogonia, which is reportedly common in xenografted testis^16,28,29^. This is because the transplanted testis initially lacked complete blood supply^8^, thereby hampering germ cell survival. Following the establishment of vascular supply from the host mice, proliferation and differentiation of the surviving germ cells was restored. However, there was subsequent increase in UCHL1 expression from 2- and 4-wk to 8-wk post-grafting, but it was still lower than that in the donor or the *in situ* control testis, which suggests incomplete recovery and persistence of initial deficit.

Elevated HSD3B and LHRH expressions at 8-wk post-grafting could be associated with a progressive increase in Leydig cell number (hyperplasia)^30^, which could not be confirmed because of the absence of stereological analysis in our study. However, these findings corroborate the recovery of seminal vesicle weight with increase in post-grafting duration in the recipient mice; in turn, this indicates restoration of serum testosterone level^8,16,19–23^. We found in an earlier study that Leydig cells could regenerate despite extensive degeneration of seminiferous tubules in ectopically autografted mice testis, leading to restoration of the weight of seminal vesicle^31^. Therefore, despite a low expression of LHR, the Leydig cells in xenografts were likely able to produce sufficient testosterone because of their increased number or enhanced steroidogenic activity.

Sertoli cells are the only somatic cells present in the seminiferous tubules that secrete growth factors (such as SCF and GDNF), express transcription regulators (such as WT1 and SOX9), and establish blood-testis barrier (BTB) in the seminiferous epithelium that are essential for germ cell development and differentiation. Presence of a steady population of Sertoli cells in xenografts was suggested by consistent expression of VIM, a structural protein and Sertoli cell-specific marker in rat testis^32^, in xenografts at all collection time points. However, precise change in cell number could not be confirmed because of the lack of stereological analysis in our study. Sertoli cells in immature rat testis were previously reported to proliferate efficiently following xenografting^8^. However, we observed dysregulation of Sertoli cell-specific proteins in our study. There was a significant decline in the expression of WT1 and SOX9. WT1 mutation has been reported to be associated with spermatogenic defects in humans^33^. Similarly, a recent study reported that ablation of SOX9 in Sertoli cells leads to complete degeneration of the seminiferous tubules^34^. We also observed elevated levels of SCF and GDNF in xenografts at an early grafting period in our study, which likely stimulated Sertoli cell proliferation, the onset of differentiation of spermatogonia, germ cell differentiation, and progression into meiosis. Previous studies have shown that SCF supports the survival of adult germ cells, onset and maintenance of spermatogenesis and germ cell differentiation and progression into meiosis^35^, and elevated levels of GDNF favors self-renewal of spermatogonia^36^. The expression of CLDN11, a transmembrane protein component of the tight junctions in the Sertoli cell BTB^37^, was found to be elevated in spermatogenic defective testis and during hypospermatogenesis, and spermatocytic and maturation arrest^38,39^. The raised CLDN11 expression in our study corroborates spermatogenic arrest in xenografts at 8-wk post-grafting.

In the current study, we assessed the endocrine status of recipients, and histological and protein expression changes in the xenografts to decipher the mechanism of spermatogenic arrest but could not perform stereological analysis. Therefore, any changes in germ and somatic cell numbers need to be further confirmed by stereological analysis, and further investigations are warranted to analyse precise cellular composition of xenografts and decipher interactions between germ and somatic cells to further understand spermatogenic arrest in xenografted testis. In conclusion, results from our study suggested that an altered hormonal milieu in recipients and dysregulated protein expression in xenografts could be a potential cause of spermatogenic arrest in xenografted immature rat testis.

## Material and Methods

### Animals and care

All animal procedures were performed in accordance with the relevant guidelines and regulations approved by the Institutional Animal Care and Use Committee (IACUC) of the Kyoto University, Japan (Permit Number 29–84, 2017). Wistar rat (*Rattus norvegicus)* pups at age 7 days post-partum were used as testis donors (n = 30). Testis from adult male rats (9-wk-old) was used as *in situ* control (n = 4), because it was demonstrated that testis xenografts have a gene expression profile similar to that of testis tissue *in situ*^40^. Immunodeficient male nude mice (CD1 Fox n1^nu^; 6–8 wk) were used as recipients (n = 15). Nude mice were kept under specific pathogen-free conditions, and food, water, and bedding were autoclaved before use. All animals used in this study were housed in 12 hours light: 12 hours darkness cycle at constant temperature, and provided food and water *ad libitum*.

### Testis collection and xenografting

Testes were collected from 7-day-old rat pups following euthanasia by CO_2_ inhalation. After collection, the epididymis was removed and testis was weighed. The tunica albuginea from immature rat testis was removed before grafting. The testes were placed on ice in Dulbecco’s modified Eagle’s medium/Ham’s F12 (DMEM/F12) HEPES (Gibco; Invitrogen, Carlsbad, CA, USA) until grafting. Four rat testes were ectopically grafted onto the back of castrated nude mice recipients. Briefly, mice were anesthetized with Avertin (2, 2, 2-tribromoethanol; 250 mg/kg body weight). For castration of recipient mice, a ventral medial incision was made in the abdomen and the testes were removed, following which the peritoneum and skin were closed using absorbable suture (Ethicon; Somerville, NJ, USA). During the same surgery, each mouse received two incisions (~5 mm) on each side of the back (four total incisions). One piece of testis was inserted through each incision. The incisions were sutured and the mice were allowed to recover and returned to their cages.

### Recipient mice analysis, recovery, and histological analysis of xenografts

The recipient mice were anesthetized as described above, and blood was collected at 2-, 4-, and 8-wk post-grafting by cardiac puncture with a heparinized syringe for hormonal assay. Serum was immediately separated by centrifugation (4,000 rpm at 4 °C) and stored at −20 °C for further analysis. For the collection of xenografts and seminal vesicles, the same recipient mice were euthanized by cervical dislocation. Xenografts were collected from recipient mice at 2-, 4-, and 8-wk post-grafting, and the percentage of xenografts recovered was calculated (Table 1). The weight of collected xenografts was also determined. All xenografts recovered at 2- and 4-wk post-grafting were used for western blot analysis. All xenografts recovered at 8-wk post-grafting were cut into two halves; one half was used for histological analysis and the other half frozen for protein isolation. The fresh donor testis from immature rats served as starting material (n = 6). The weight of the seminal vesicle from recipient mice at 2-, 4-, and 8-wk post-grafting (n = 5 for each time point) was measured as an indicator of bioactive testosterone production by the grafted tissue^19,20,41^. The average weight of seminal vesicle from non-grafted intact adult nude male mice (CD1 Fox n1^nu^; 6–8 wk, n = 8) was taken as control.

For histological analysis, donor testes (n = 3), xenografts collected at 8-wk post-grafting (19 xenografts recovered from 5 donors), and adult *in situ* control testis (n = 4) tissues were fixed in Bouin’s solution followed by tissue dehydration with increased concentrations of alcohol in a series. After processing, the tissues were embedded in paraffin, sectioned (7-µm thick), stained with hematoxylin and eosin (H&E), dehydrated, mounted in Vectamount (Vector Laboratories; Burlingame, CA, USA), and observed under a light microscope (Nikon; Melville, NY, USA). Establishment of spermatogenesis was assessed by morphological evaluation, as previously described^14^. A xenograft without distinct cell types and tubules was classified as either degenerated or non-degenerated if it contained even a single seminiferous tubule. More than 40 seminiferous tubule cross-sections were examined randomly in a xenograft to investigate the status of spermatogenesis. The most advanced germ cell type and differentiated germ cells were identified by their morphology and location in the seminiferous tubule. The xenografts with evidence of spermatogenesis (17 out of 19 recovered xenografts) were further used for data analysis. The percentage of tubules with the most advanced germ cell type was presented. The percentage of seminiferous tubules and degenerated tubules within a graft were also presented. The percentage of seminiferous tubules and degenerated tubules within a graft was also calculated.

### Hormonal assay

Serum levels of FSH and LH were determined using ELISA kits (LS Bio, Seattle, WA, USA) according to the manufacturer’s instructions. All samples were tested in triplicate. The sensitivities of the mouse FSH and LH assays were 0.313 ng/ml and 0.31 mIU/ml, respectively.

### Western blotting analysis of xenografts, donor and *in situ* control testes

The xenografts were analyzed for the expression of testis-specific proteins by western blot analysis. Total protein from all xenografts recovered at 2- and 4-wk post-grafting and xenografts with meiotic germ cells collected at 8-wk post-grafting was extracted upon homogenization by sonication in a dissolving buffer (7 M urea, 2 M thiourea, 4% CHAPS, 18 mMTris–HCl, 14 mMTris– Base, 0.2% Triton-X, and 50 mM dithiothreitol). Single-strength ProteCEASE-50, EDTA-free protease inhibitor (G-Biosciences, St. Louis, MO, USA) was added to the dissolving buffer before protein extraction. All xenografts collected at 2-, 4-, and 8-wk post-grafting from a recipient were pooled for protein isolation (n = 5 for each collection time point). Protein sample from pooled xenografts, at a given collection time point (2-, 4-, and 8-wk post-grafting) from at least 4 recipients was used for western blot analysis. Pooled proteins from the testis of 7-day-old rat donors (n = 5) represented the starting material and 9-wk-old rats were used as *in situ* control (n = 4). Protein concentration was determined by the Bradford method using a Bio-Rad protein assay kit (Bio-Rad Laboratories, Hercules, CA, USA). The lysed samples (40 µg) were subjected to electrophoresis on 8% (for FSHR, LHR, and AR antibodies), 10% (for HSD3B, VIM, WT1, SOX9, SCF, INHBB, and ACTIN antibodies), or 12% (for UCHL1, CLDN11, and GDNF antibodies) SDS–polyacrylamide gel. The gels were transferred onto PVDF membranes (Millipore; Billerica, MA, USA). The membranes were blocked with Starting Block TBS blocking buffer (Thermo Scientific; Waltham, MA, USA) for 1 h at room temperature. The blocked membranes were incubated with one of the following primary antibodies overnight at 4 °C: UCHL1 (sc-271639, 1:100), HSD3B (sc-51520, 1:200), SOX9 (sc-20095, 1:200), CLDN11 (sc-271232, 1:200), SCF (sc-13126, 1:200), ACTB (sc-47778, 1:200), INHBB (sc-376971, 1:100), FSHR (sc-7798, 1:200) (all from Santa Cruz Biotechnology; Dallas, TX, USA), WT1 (PA1-20991, 1:500), GDNF (PA1-9524, 1:2000), LHR (PA5-21271, 1:100), AR (PA5-16363, 1:100) (all from Thermo Scientific), and VIM (M0725, 1:5000, Dako, Agilent Technologies Japan, Ltd., Tokyo, Japan). The membranes were then washed with TBS-T and incubated with goat anti-rabbit, goat anti-mouse, or rabbit anti-goat HRP-conjugated secondary antibody (1:10000; all from Thermo Scientific) in TBS-T for 1 h at room temperature. After washing with TBS-T, immunoreactivity was detected by chemoluminescence using Hyperfilm ECL (GE Healthcare Japan Corporation, Tokyo, Japan) against ECL™ Prime chemiluminescent substrate (GE Healthcare). To control protein loading on the gels, the membranes were stripped, blocked and further probed with ACTB antibody.

Immunoreactivity of blots was detected by chemoluminescence using Hyperfilm ECL (GE Healthcare Japan Corporation) against ECL™ Prime chemiluminescent substrate (GE Healthcare). Hyperfilms were scanned in high resolution, and digital images were exported to Image Lab™ Software Version 6.0.1 (Bio-Rad Laboratories) for image densitometric analysis. The densitometric value for a given antibody was normalized with densitometric value of ACTB (loading control) for each lane in every blot. To generate the graphs and statistical analysis, normalized densitometry data from at least 4 blots were considered.

### Statistical analyses

The results were presented as mean ± S.E.M. The statistical analyses were performed by ANOVA. Normality and homogeneity of variances were checked and confirmed to match with parametric assumptions prior to running ANOVA. Significant differences between the means were determined by analyzing the data using the Tukey honest significance difference (Tukey HSD) test. The level of significance was set at P < 0.05.

## Supplementary information

Supplementary file 1

## Data Availability

All data generated or analyzed during this study are included in this published article (and its Supplementary Information files).
