# Supplementary material for: Altered hormonal milieu and dysregulated protein expression can cause spermatogenic arrest in ectopic xenografted immature rat testis
Source: Sci Rep. 2019 Mar 11;9:4036. doi: 10.1038/s41598-019-40662-y (PMC6411886; doi:10.1038/s41598-019-40662-y)

Title: Altered hormonal milieu and dysregulated protein expression can cause spermatogenic arrest in ectopic xenografted immature rat testis

Authors: Sandeep Goel[1, 2] * and Naojiro Minami[1]

Affiliation: [1]Laboratory of Reproductive Biology, Graduate School of Agriculture, Kyoto University, Kyoto 606-8502, Japan

[2]Laboratory for the Conservation of Endangered Species, Centre for Cellular and Molecular Biology, Council for Scientific and Industrial Research, Uppal Road, Hyderabad, 500 007, India

*Corresponding author:

Sandeep Goel (goel.sandeep.6r@kyoto-u.ac.jp)

Supplementary table 1: Analysis of xenografts for tubules containing most advanced germ cells

(Presented in figure 2E)

Recipient #	Xenograft#	Number of tubules counted	Tubules containing most advanced germ cells						
			Gon/Spg	Spermatocyte	Rd Spd	Elon Spd	Spermatozoa	SC	Deg Tubules
1	1.1	54	13	31	0	0	0	8	2
	1.2	60	11	37	0	0	0	7	5
	1.3	43	9	26	0	0	0	5	3
2	2.1	59	12	36	0	0	0	9	2
	2.2	57	8	36	0	0	0	11	2
	2.3	45	8	27	0	0	0	6	4
	2.4	54	14	30	0	0	0	7	3
3	3.1	63	10	41	0	0	0	8	4
	3.2	61	14	37	0	0	0	8	2
	3.3	69	18	42	0	0	0	4	5
4	4.1	48	9	30	0	0	0	5	4
	4.2	52	14	29	0	0	0	7	2
	4.3	44	11	26	0	0	0	5	2
	4.4	43	14	17	0	0	0	9	3
5	5.1	68	15	40	0	0	0	8	5
	5.2	52	11	29	0	0	0	8	4
	5.3	62	10	42	0	0	0	7	3
Total		934	201	556	0	0	0	122	55

Deg tubules, degenerated tubules; SC, Sertoli cell only; Gon/spg, gonocytes or spermatogonia; Spcyt, pachytene spermatocytes; Rd Spd, round spermatid; Elon Spd, elongated spermatid; Spermatozoa, spermatozoa

Supplementry Fig.1: Full images of western blots

Fig. 3

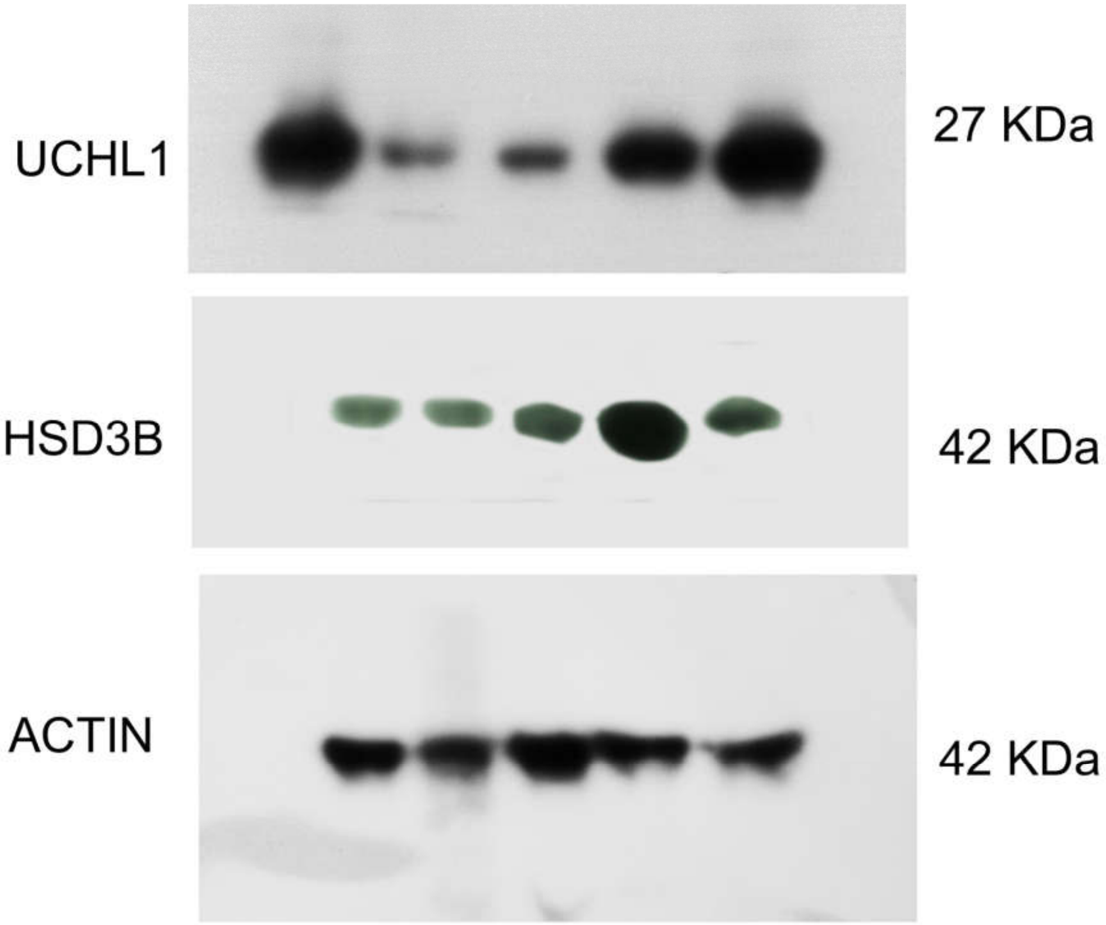

Supplementry Fig. 2: Full images of western blots

Fig. 4

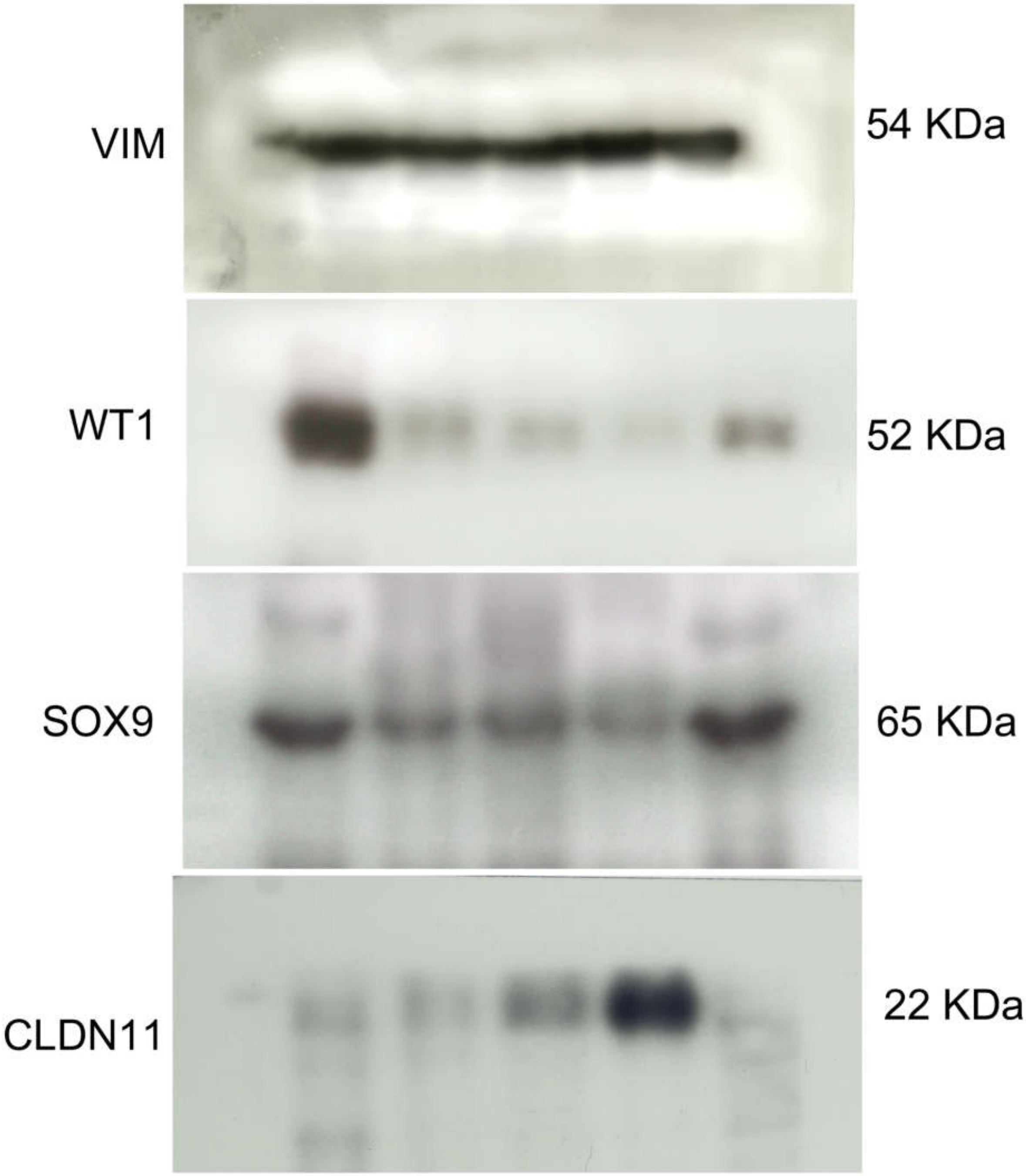

Supplementry Fig. 3: Full images of western blots
Fig. 5

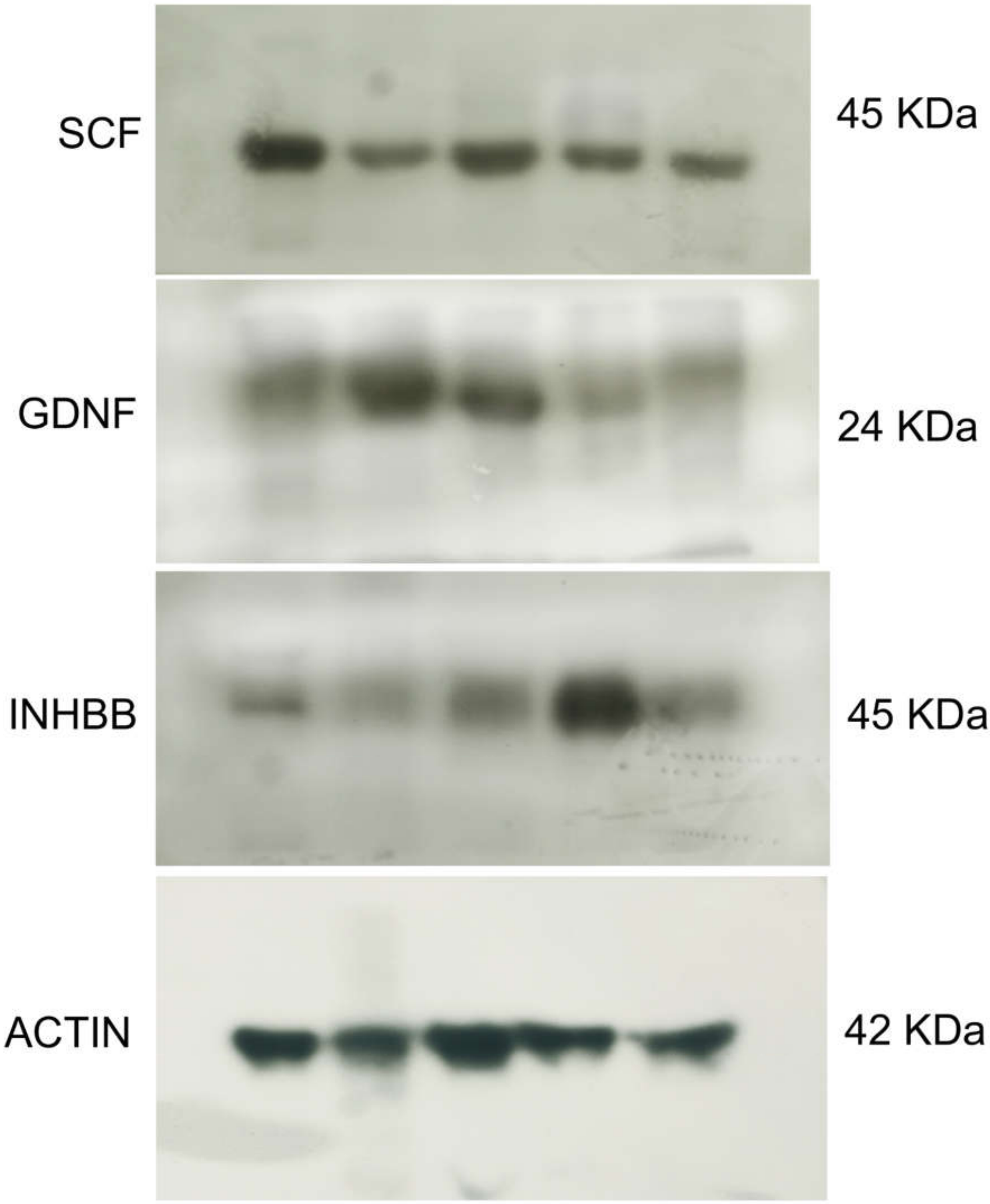

Supplementry Fig. 4: Full images of western blots

Fig. 6

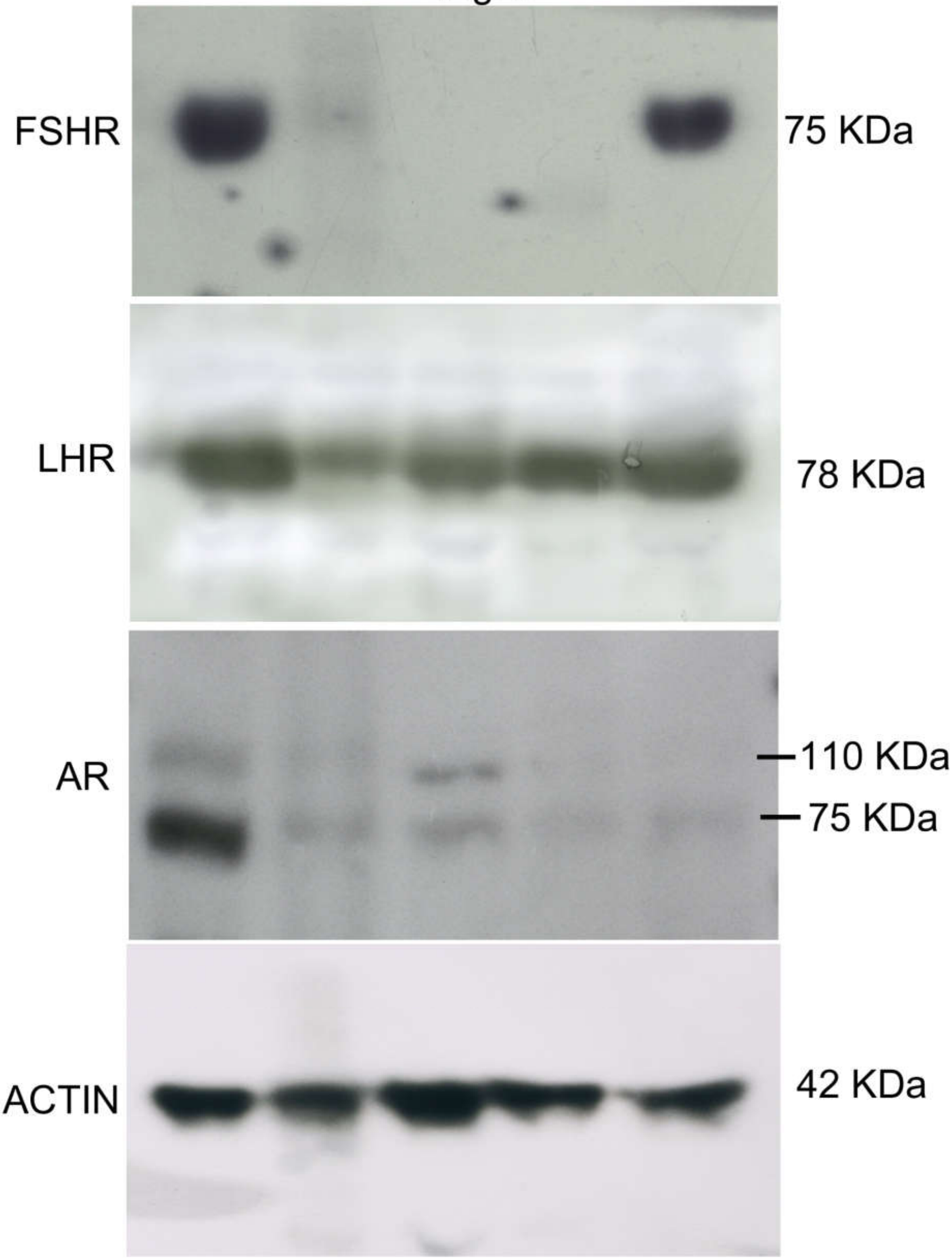

Supplement: Supplementary file 1 [file 41598_2019_40662_MOESM1_ESM.pdf]
